# Major depression among pregnant women attending a tertiary teaching hospital in Northern Uganda assessed using DSM-V criteria

**DOI:** 10.1186/s12884-025-07618-9

**Published:** 2025-04-26

**Authors:** Jerom Okot, Henry Ochola, Nozuko P. Blasich, Michael Job Aeku, Pebalo Francis Pebolo, Felix Bongomin, Simple Ouma

**Affiliations:** 1 Faculty of Medicine, Gulu University, Gulu, Uganda; 2 Faculty of Epidemiology and Population Health, London School of Hygiene and Tropical Medicine, London, UK; 3 Academic Affairs, Research and Quality Assurance National Health Laboratory Service, Johannesburg, South Africa; 4 Department of Sexual and Reproductive Health, Gulu University, Gulu, Uganda; 5 Department of Medical Microbiology and Immunology, Faculty of Medicine, Gulu University, Gulu, Uganda; 6 Department of Internal Medicine, Gulu Regional Referral Hospital, Gulu, Uganda; 7 Department of Public Health, Gulu University, Gulu, Uganda; 8 The AIDS Support Organization, Kampala, Uganda

**Keywords:** Major depression, Antenatal care, Pregnancy

## Abstract

**Background:**

Major depression (MD) during pregnancy complicates maternal and neonatal outcomes. Despite its significant impact, there is a lack of evidence on the prevalence and associated factors of MD among pregnant women in Uganda. We assessed the magnitude and factors associated with MD among pregnant women attending antenatal care (ANC) at a large teaching hospital in Northern Uganda.

**Methods:**

Between June and August 2023, we enrolled pregnant women aged 18 years or older attending ANC clinic at Gulu Regional Referral Hospital in Northern Uganda. Data were collected using a validated semi-structured questionnaire. MD was evaluated using DSM-V criteria and was defined as having (1) at least two weeks of either persistent low mood or excessive sadness, (2) plus additional symptoms from the MD diagnostic criterion A, for a total of at least five MD symptoms, and (3) the symptoms caused significant distress or problem and significantly altered behaviour or functionality. Modified Poisson regression analyses with robust standard errors was constructed to evaluate for factors independently associated with major depression. Adjusted prevalence ratio (aPR) whose 95% confidence interval (CI) did not include the null value (0) or *p* < 0.05 was considered statistically significant.

**Results:**

We enrolled 329 participants, with a mean age of 26.1± 5.5 years. Overall, 29.8% (*n* = 98) had MD; 37 (11.2%) mild, 49 (14.9%) moderate, and 12 (3.6%) severe MD. Having a co-wife (aPR: 1.64, 95% CI:1.09–2.45, *p* = 0.016), an arranged marriage (aPR: 1.56, 95% CI: 1.02–2.42, *p* = 0.042), partner’s income in second quartile (aPR: 2.14, 95% CI: 1.29–3.54, *p* = 0.003), experiencing physical violence (aPR: 1.75, 95% CI: 1.09–3.81, *p* = 0.019), controlling behaviours from partner (aPR: 3.60, 95% CI: 1.79–7.26, *p* < 0.001), and planned pregnancy (aPR: 0.53%, 955 CI: 0.35–0.81, *p* = 0.003) were independently associated with MD.

**Conclusion:**

Major Depression affects nearly one-third of pregnant women in Northern Uganda. Major Depression is more prevalent among women with co-wives, in arranged marriages, with unplanned pregnancies, whose partners had low income, who experienced physical violence or controlling behaviours from a partner. These findings highlight the urgent need for targeted interventions, including prevention, screening, and treatment services for Major Depression within Antenatal Care clinics. Implementing such measures is crucial to improving maternal, foetal, and neonatal health outcomes in the region.

**Supplementary Information:**

The online version contains supplementary material available at 10.1186/s12884-025-07618-9.

## Background

Maternal mental health remains major public health concerns accounting for over 10% among pregnant women and 13% among women who have just given birth [1]. However, in low in-come countries and developing countries, the burden of depression is up to 15.6% during pregnancy and 19.8% after childbirth [2]. Nearly every woman faces the possibility of developing mental disorders during pregnancy and within the first year after giving birth [1, 3, 4]. However, the risk of specific disorders tends to rise in cases of poverty, migration, severe stress, exposure to violence (including domestic, sexual, economical, emotional, physical and controlling behaviours), emergencies, conflicts, natural disasters, and lack of social support [3, 5–7]. Depression during pregnancy can range from mild to severe form [3, 8].

Global burden of antenatal depression to range from 15 to 65% worldwide with associated risk factors such as exposure to violence, lack of partner or social support, personal or family history of mental disorders [9]. A systematic review of studies conducted in Africa reports that, 26.3% of pregnant women experience antenatal depression, particularly those in economic difficulties, unfavourable marriage conduction, non-support from relatives and bad obstetric history [10]. Major depression is associated with substance abuse, decreased utilization of healthcare services, poor appetite and suicidal thoughts in some extreme cases, it may even lead to suicide among pregnant women however they are also at risk of bleeding during pregnancy, miscarriage, and hypertensive disorder [3, 11]. Babies born to mother who was depressed during the pregnancy are at risk of restricted growth, low birth weight, low Apgar scores and admission to neonatal intensive care units [4].

In Uganda, a study conducted at Mubende Regional Referral Hospital, reports 37.68% prevalence of antenatal depression among pregnant women [12]. Also, another study done in Lira district northern Uganda reports prevalence of 36.1% in early pregnancy [13]. Depression in pregnancy is the most common mental health issue among pregnant women, it often goes unnoticed and untreated, potentially resulting in negative outcomes for both mothers and babies [13–15]. In Gulu Northern Uganda, where the healthcare landscape is marked by post-conflict recovery and ongoing socio-economic challenges, understanding the prevalence and associated factors of Major Depression (MD) among pregnant women is crucial for targeted intervention strategies and improving the mental well-being of mothers during Antenatal Care (ANC) hence positive outcome to both the mother and the baby. We determined the prevalence and factors associated with MD in pregnancy among women in northern Uganda.

## Methods

### Study design

This facility-based cross-sectional study was conducted at the ANC clinic at Gulu Regional Referral Hospital (GRRH).

### Study setting

GRRH is a tertiary hospital for patients from mid-northern Uganda, thus serving both rural and urban populations within the catchment area. GRRH also receives patients from neighbouring countries of South Sudan and the Democratic Republic of Congo. It is also a teaching hospital for the Gulu University, Trainer Centre for Fellowship with East Central and Southern African College of Obstetrics and Gynaecology (ECSACOG) and many other Diploma and Certificate Training Institutions in the region. It is a hospital with both out and in-patient services for an estimated 120,000 patients per year. The hospital has specialised units managed by consultants from GRRH and the Faculty of Medicine, at Gulu University. The Obstetrics and Gynaecology department provides all services ranging from ANC to Postnatal Care (PNC) during pregnancy and after delivery. Due to the quality and free ANC services, a large proportion of pregnant women within the catchment area attend their ANC services at GRRH.

### Study population

This study was conducted among pregnant women attending ANC at GRRH. All pregnant women attending ANC at GRRH were eligible for inclusion, irrespective of gestational age. However, we intended to exclude pregnant women who were deemed unable to provide informed consent due to severe illness or mental disorder. However, no pregnant women were found to meet the exclusion criteria.

### Sample size

Using GPower, the minimum sample size was estimated to be 301. Assuming.

a conservative non-response rate of 10% (301/0.9), the adjusted sample size was 335 participants.

### Sampling techniques

Based on the average daily attendance at the ANC clinic at GRRH which was approximately 80 women per day, we used a systematic random sampling method aided by the daily ANC attendance register.

### Data collection tool and process

Data were collected from 329 pregnant women attending their ANC at GRRH between June and August 2023 by trained research assistance under supervision of the principal investigator. We selected every 5th person on the list on any given day. This enables each of the four investigators who participated in data collection to handle at least four participants per day. While on the ground the daily ANC attendance ranged between 60 and 100 pregnant women and thus, we collected data from between 12 and 20 participants on any given day. Data were collected using a semi-structured questionnaire which was developed for this study by the authors. Originally, the tool was developed in English and later translated into Acholi language (Luo) and then pre-tested, for consistency in question interpretation and language appropriateness, among 20 pregnant women attending ANC at Laroo Health Centre III. Ac copy of the English version of questionnaire has been uploaded as supplementary file. Data were collected using interviewer-administered questionnaires in either Acholi or English language depending on the participant’s literacy level and preferences.

### Study variables

Independent variables included socio-demographic characteristics like age, education, being in a union, employment status, partner level of education, partner employment status, age at first sex, age at first marriage, age of the pregnancy in weeks and, living with HIV and partner alcohol use. The outcome variable for this study was MD. Participants with MD were those who met the DSM-5 diagnostic criteria for MD, (1) at least 2 weeks of either persistent low mood or excessive sadness, (2) plus additional symptoms from the MD diagnostic criterion A, for a total of at least five MD symptoms, and (3) the symptoms caused significant distress or problem and significantly altered their behaviour or functionality.

### Data management and statistical analysis

Data were entered and cleaned in Microsoft Excel 2023. The cleaned data was coded and then exported to STATA 17.0 for analysis. Descriptive statistics were summarised using frequency with corresponding proportions for categorical variables or mean with corresponding standard deviations (SD) for numerical variables with normal distribution and median plus inter-quartile range for numerical variables with skewed distributions. To examine the associations between independent factors and MD, we conducted univariable and multivariable modified Poisson regression analyses with robust standard errors. We reported univariable analyses using unadjusted prevalence ratios (uPR) with corresponding confidence intervals (CI) and *p*-value. All independent variables with *p* < 0.20 at univariable analysis were considered for inclusion into the multivariable modified Poisson regression model. Since they have a study power of greater than 80%. The model was built using backward elimination method with best fit model selected using AIC. The results from the multivariable model were reported using the adjusted Prevalence ratios (aPR) with corresponding 95% CIs and *p*-values. Any *p* < 0.05 or effect size whose CI did not include the null value (0) was considered statistically significant.

## Results

### Baseline characteristics of the participants

We included 329 participants in the final analysis. Overall mean age was 26.06 (SD: 5.52) years. Majority were from within Gulu (89.8%, *n* = 281), in union (90.0%, *n* = 296), with negative HIV status (91.55, *n* = 301), partners employed (80.8%, *n* = 265), were in love marriage (81.5%, *n* = 247), and had a co-wife (76.0%, *n* = 247). More than half were not employed (54.1%, *n* = 178), ever used contraceptive (57.8%, *n* = 188), had wanted pregnancy (65.3%, *n* = 215), and husband doesn’t drink alcohol (57.2%, *n* = 187). 66(20.2%) had attained tertiary level of education, 8%(*n* = 26) A level, 32.8%(*n* = 107) O level and 28.2%(*n* = 92) upper primary. More than one third of the participants and their partners had third or highest income quartile, 44.3%(*n* = 70) and 46.7%(*n* = 115) respectively. The average age at first sex and first marriage were 17.81(SD:2.46) and 20.36(SD:3.27) respectively. Mean number of children was 1.59(SD:1.36) and mean week of amenorrhoea was 23.11(SD: 8.96) weeks. (Table 1), describe the characteristics of the participants.

**Table 1:** Baseline characteristic of the participants

Variable	Major depression		All (*N* = 329) *n* (%)
	No (***n* = 231)** *n* (%)	Yes (***n* = 98)** *n* (%)	
Place of residence, *n* = 313			
Gulu Other districts	191(87.3) 28(12.7)	89(95.7) 4(4.3)	281(89.8) 32(10.2)
Education level of the participant			
Lower primary or none Upper primary O level A level Tertiary	21(9.2) 62(27.2) 73(32.0) 15(6.6) 57(25.0)	14(14.3) 30(30.6) 34(34.7) 11(11.2) 9(9.2)	35(10.7) 92(28.2) 107(32.8) 26(8.0) 66(20.2)
Marital status			
Not in union In union	16(6.9) 215(93.1)	17(17.3) 81(82.7)	33(10.0) 296(90.0)
HIV status			
Negative Positive	215(93.1) 16(6.9)	86(87.8) 12(12.2)	301(91.5) 28(8.5)
Has a co wife			
No Yes	187(82.0) 41(18.0)	60(61.9) 37(38.1)	247(76.0) 78(24.0)
Employment status			
Not employed Employed	118(51.1) 113(48.9)	60(61.2) 38(38.8)	178(54.1) 151(45.9)
Employment status of the partner			
Not employed Employed	42(18.3) 188(81.7)	21(21.4) 77(78.6)	63(19.2) 265(80.8)
Education level of the partner			
Lower primary or none Upper primary O level A level Tertiary	10(4.3) 24(10.4) 62(27.0) 37(16.1) 97(42.2)	8(8.2) 4(4.1) 37(37.8) 21(21.4) 28(28.6)	18(5.5) 28(8.5) 99(30.2) 58(17.7) 125(38.1)
Ever used contraceptive			
No Yes	93(40.6) 136(59.4)	44(45.8) 52(54.2)	137(42.2) 188(57.8)
Wanted pregnancy			
No Yes	69(29.9) 162(70.1)	45(45.9) 53(54.1)	114(34.7) 215(65.3)
Pregnancy was wanted by husband			
Husband or both Wife alone	214(93.4) 15(6.6)	72(76.6) 22(23.4)	286(88.5) 37(11.5)
Marriage type, *n* = 303			
Love marriage Arranged marriage	183(83.9) 35(16.1)	64(75.3) 21(24.7)	247(81.5) 56(18.5)
Relationship with family members of the partner			
Very good Good Poor or fair	75(32.9) 109(47.8) 44(19.3)	25(25.8) 30(30.9) 42(43.3)	100(30.8) 139(42.8) 86(26.5)
Husband drinks alcohol			
No Yes	141(61.3) 89(38.7)	46(47.4) 51(52.6)	187(57.2) 140(42.8)
Quartile of income			
Lowest quartile Second quartile Third/ highest quartile	28(24.1) 32(27.6) 91(52.3)	18(42.9) 10(23.8) 14(33.3)	46(29.1) 42(26.6) 70(44.3)
Quartile of income of the partner			
Lowest quartile Second quartile Third/ highest quartile	47(27.0) 36(20.7) 91(52.3)	16(22.2) 32(44.4) 24(33.3)	63(25.6) 68(27.6) 115(46.7)
Physical violence			
Yes No	51(22.1) 180(77.9)	40(40.8) 58(59.2)	91(27.7) 238(72.3)
Sexual violence			
Yes No	55(23.8) 176(76.2)	60(61.2) 38(38.8)	115(35) 214(65.0)
Emotional violence			
Yes No	42(18.2) 189(81.8)	50(51.0) 48(49)	92(28) 237(72.0)
Economical			
Yes No	119(51.5) 112(48.5)	82(83.7) 16(16.3)	201(61.1) 128(38.9)
Controlling behaviours			
Yes No	20(8.7) 211(91.3)	32(32.7) 66(67.3)	52(15.8) 277(84.2)
Multiple GBV			
Yes No	84(36.4) 147(63.6)	70(71.4) 28(28.6)	154(46.8) 175(53.2)
	**Mean (SD)**	**Mean (SD)**	**Mean (SD)**
Age in years	26.19(5.23)	25.76(6.16)	26.06(5.52)
Age at first sexual intercourse (years)	17.93(2.24)	17.54(2.91)	17.81(2.46)
Age of first marriage (years)	20.41(3.43)	20.23(2.85)	20.36(3.27)
Number of children at home	1.56(1.28)	1.67(1.54)	1.59(1.36)
Week of amenorrhoea (weeks)	23.31(9.02)	22.67(8.85)	23.11(8.96)

### Prevalence of major depression among the participants

Overall, 98/329 (29.8%) had major depression. Half of the participants had moderate depression (50%, *n* = 49), 12.2%(*n* = 12) had severe depression, and 37 (37.8%) had mild depression. (Table 2)

**Table 2:** Prevalence of major depression in pregnancy among participants

DSM V diagnostic Criteria for MD (*N* = 329)	Yes, *n* (%)
1. Have you been consistently depressed or down, most of the day, nearly every day, for the past two weeks?	113(47.2)
2. In the past two weeks, have you been much less interested in most things or much less able to enjoy the things you used to enjoy most of the time?	113 (34.4)
3. Was your appetite decreased or increased nearly every day? Did your weight decrease or increase without trying intentionally?	145 (44.1)
4. Did you have trouble sleeping (difficulty falling asleep, waking up in the middle of the night, early morning awakening, or sleeping excessively) nearly every night?	143 (43.5)
5. Did you talk or move more slowly than normal, or were you fidgety, restless, or having trouble sitting still almost every day?	97 (29.5)
6. Did you feel tired or without energy almost every day?	159 (48.3)
7. Did you feel worthless or guilty almost every day?	83 (25.2)
8. Did you have difficulty concentrating or making decisions almost every day?	92 (28.0)
9. Did you repeatedly consider hurting yourself, feel suicidal, or wish that you were dead?	250 (17.9)
10. Do symptoms mentioned above cause significant distress or problems at home, at work, socially, in your relationships, or in some other way, and are they a change from previous functioning?	153 (28.0)
Participant meet*s* the *DSM-5 diagnostic criteria for MD*	98 (29.8)
**Severity of MD** (***n*** = 98)	
Mild (had five MD symptoms)	37 (37.8)
Moderate (had 6–7 MD symptoms)	49 (50.0)
Severe (had 8–9 MD symptoms)	12 (12.2)

### Factors significantly associated with major depression in pregnancy among the participants

At simple Poisson regression, factors positively associated with major depression were having a co-wife (cPR: 1.95, 95% CI: 1.42–2.69, *p* < 0.001),, pregnancy wanted by wife alone(cPR: 2.36, 95% CI: 1.69–3.29, *p* < 0.001), having poor or fair relationship with partner’s family(cPR: 1.95, 95% CI: 1.30–2.92, *p* = 0.001), husband drink alcohol (cPR: 1.48, 95% CI: 1.06–2.07,*p* = 0.021), partner’s income quartile being second (cPR: 1.85, 95% CI: 1.13–3.04, *p* = 0.014), exposed to physical violence(cPR: 2.6, 95% CI: 1.91–3.49,*p* < 0.0001), sexual violence(cPR: 1.8, 95% CI: 1.30–2.49, *p* < 0.0001), emotional violence(cPR: 2.9, 95% CI: 1.30–2.49, *p* < 0.0001), economical violence(cPR: 2.7, 95% CI: 2.09–4.12), controlling behaviours (cPR: 3.3, 95% CI: 2.00-5.32, *p* < 0.0001) and multiple GBV(cPR: 2.8, 95% CI: 1.94–4.16, *p* < 0.0001). Factors negatively associated with major depression were participant’s income quartile of third or highest quartile (cPR: 0.51, 95% CI: 0.28–0.92, *p* = 0.027), wanted pregnancy(cPR:0.62, 95% CI: 0.45–0.87, *p* = 0.005), having attained tertiary level of education (cPR: 0.34, 95% CI: 0.16–1.004, *p* = 0.004), and being in union (cPR: 0.54, 95% CI:0.36–0.78, *p* = 0.001). (Table 3)

### Factors independently associated with major depression in pregnancy among the participants

Factors independently associated positively with major depression among the participants were having co-wife(aPR: 1.64, 95% CI:1.09–2.45, *p* = 0.016), being in arranged marriage(aPR: 1.56, 95% CI: 1.02–2.42, *p* = 0.042), partner’s income quartile of second quartile (aPR: 2.14, 95% CI: 1.29–3.54, *p* = 0.003), experiencing physical violence (aPR: 1.75, 95% CI: 1.09–3.81, *p* = 0.019) and controlling behaviours from partner (aPR: 3.60, 95% CI: 1.79–7.26, *p* < 0.001). However, having wanted pregnancy was protective of major depression among the participants (aPR: 0.53%, 955 CI: 0.35–0.81, *p* = 0.003), Table 3.

**Table 3:** Factors associated with major depression in pregnancy among the participants

Variable	Major depression		Crude PR (95% CI)	*P* value	Adjusted PR (95% CI)	*P* value
	No (*n* = 231) *n* (%)	Yes (*n* = 98) *n* (%)				
Place of residence						
Gulu Other districts	191(87.3) 28(12.7)	89(95.7) 4(4.3)	Reference 0.39(0.16–1.004)	0.051	---	---
Education level of the participant						
Lower primary or none Upper primary O level A level Tertiary	21(9.2) 62(27.2) 73(32.0) 15(6.6) 57(25.0)	14(14.3) 30(30.6) 34(34.7) 11(11.2) 9(9.2)	Reference 0.82(0.49–1.35) 0.79(0.49–1.29) 1.05(0.58–1.93) 0.34(0.16–0.71)	0.425 0.360 0.856 0.004	---	---
Marital status						
Not in union In union	16(6.9) 215(93.1)	17(17.3) 81(82.7)	Reference 0.53(0.36–0.78)	0.001	---	---
Marriage type						
Love marriage Arranged marriage	183(83.9) 35(16.1)	64(75.3) 21(24.7)	Reference 1.45(0.97–2.16)	0.069	Reference 1.56(1.02–2.42)	0.042
HIV status						
Negative Positive	215(93.1) 16(6.9)	86(87.8) 12(12.2)	Reference 1.5(0.94–2.39)	0.087	---	---
Has a co wife						
No Yes	187(82.0) 41(18.0)	60(61.9) 37(38.1)	Reference 1.95(1.42–2.69)	< 0.0001	Reference 1.64(1.09–2.45)	0.016
Employment status						
Not employed Employed	118(51.1) 113(48.9)	60(61.2) 38(38.8)	Reference 0.75(0.53–1.05)	0.096	---	---
Employment status of the partner						
Not employed Employed	42(18.3) 188(81.7)	21(21.4) 77(78.6)	Reference 0.87(0.59–1.29)	0.498	---	---
Education level of the partner						
Lower primary or none Upper primary O level A level Tertiary	10(4.3) 24(10.4) 62(27.0) 37(16.1) 97(42.2)	8(8.2) 4(4.1) 37(37.8) 21(21.4) 28(28.6)	Reference 0.32(0.11–0.91) 0.84(0.47–1.49) 0.81(0.44–1.51) 0.50(0.27–0.93)	0.033 0.556 0.517 0.028	---	---
Ever used contraceptive						
No Yes	93(40.6) 136(59.4)	44(45.8) 52(54.2)	Reference 0.86(0.62–1.21)	0.384	---	---
Wanted pregnancy						
No Yes	69(29.9) 162(70.1)	45(45.9) 53(54.1)	Reference 0.62(0.45–0.87)	0.005	Reference 0.53(0.35–0.81)	0.003
Pregnancy was wanted by husband						
Husband or both Wife alone	214(93.4) 15(6.6)	72(76.6) 22(23.4)	Reference 2.36(1.69–3.29)	< 0.001	---	---
Relationship with family members of the partner						
Very good Good Poor or fair	75(32.9) 109(47.8) 44(19.3)	25(25.8) 30(30.9) 42(43.3)	Reference 0.86(0.541.37) 1.95(1.30–2.92)	0.536 0.001	---	---
Husband drinks alcohol						
No Yes	141(61.3) 89(38.7)	46(47.4) 51(52.6)	Reference 1.48(1.06–2.07)	0.021	---	---
Quartile of income						
Lowest quartile Second quartile Third/ highest quartile	28(24.1) 32(27.6) 91(52.3)	18(42.9) 10(23.8) 14(33.3)	Reference 0.61(0.32–1.17) 0.51(0.28–0.92)	0.135 0.027	---	---
Quartile of income of the partner						
Lowest quartile Second quartile Third/ highest quartile	47(27.0) 36(20.7) 91(52.3)	16(22.2) 32(44.4) 24(33.3)	Reference 1.85(1.13–3.04) 0.82(0.47–1.43)	0.014 0.487	Reference 2.14(1.29–3.54) 1.07(0.58–1.96)	0.003 0.836
Physical violence						
Yes No	51(22.1) 180(77.9)	40(40.8) 58(59.2)	2.6(1.91–3.49) Reference	< 0.0001	1.75(1.09–3.81) Reference	0.019
Sexual violence						
Yes No	55(23.8) 176(76.2)	60(61.2) 38(38.8)	1.8(1.30–2.49) Reference	< 0.0001	---	---
Emotional violence						
Yes No	42(18.2) 189(81.8)	50(51.0) 48(49)	2.9(2.09–4.12) Reference	< 0.0001	---	---
Economical						
Yes No	119(51.5) 112(48.5)	82(83.7) 16(16.3)	2.7(1.96–3.68) Reference	< 0.0001	---	---
Controlling behaviours						
Yes No	20(8.7) 211(91.3)	32(32.7) 66(67.3)	3.3(2.00-5.32) Reference	< 0.0001	3.60(1.79–7.26) Reference	< 0.001
Multiple GBV						
Yes No	84(36.4) 147(63.6)	70(71.4) 28(28.6)	2.8(1.94–4.16) Reference	< 0.0001	---	---
	Mean (SD)	Mean (SD)	Mean (SD)		---	---
Age in years	26.19(5.23)	25.76(6.16)	0.99(0.96–1.02)	0.550	---	---
Age at first sexual intercourse (years)	17.93(2.24)	17.54(2.91)	0.96(0.89–1.03)	0.221	---	---
Age of first marriage (years)	20.41(3.43)	20.23(2.85)	0.99(0.94–1.03)	0.628	---	---
Number of children at home	1.56(1.28)	1.67(1.54)	1.04(0.91–1.19)	0.548	---	---
Week of amenorrhoea (weeks)	23.31(9.02)	22.67(8.85)	0.99(0.98–1.01)	0.554	---	---

## Discussion

Our study report that 29.8% of pregnant women had MD. Factors such as having a co-wife increased the prevalence of MD by 64% compared to not having a co-wife, being in an arranged marriage increased the prevalence of MD by 56% compared to love marriage, and being in the second quartile of partner’s income increased the prevalence of MD by 114% compared to the lowest quantile. Additionally, experiencing physical violence increased the likelihood by 75% while experiencing controlling behaviours from a partner increased it by 260%. Conversely, having a wanted pregnancy was found to be protective against major depression, reducing the prevalence by 46% among women with wanted compared to women with unwanted pregnancy.

This study found that the prevalence of MD is 29.8%. This finding is consistence research conducted in Africa [9]. However, some studies have reported higher prevalence of MD among pregnant women. For example a study done in central Uganda (12,). Some study also reports relatively lower prevalence of depression in pregnancy [6, 16–21]. Similarly, a study conducted in post-COVID-19 China and delta region in Nigeria also reported a comparable prevalence [22, 23]. These differences in prevalence suggest that regional and other factors including cultural attitudes, diagnostic practices, and available support systems, significantly influence reported prevalence. This variability highlights the need for localized studies and tailored mental health interventions to effectively address antenatal depression.

Having a co-wife increased the prevalence of MD by 64% compared to not having a co-wife. This aligns with previous research indicating that marital conflict and competition among wives can contribute to increased psychological stress and depression among pregnant women.

Similarly, being in an arranged marriage increased the prevalence of MD by 56% compared to love marriage. Arranged marriages may pose challenges related to marital adjustment, autonomy, and emotional support, which are crucial factors in maternal mental health during pregnancy.

Financial stability, as reflected by the partner’s income quartile, also emerged as a significant factor. Women whose partners fell into the second quartile of income were 2.14 times more likely to experience major depression. Financial stressors can exacerbate psychological distress during pregnancy, impacting maternal mental health negatively.

The study also highlighted the impact of physical violence on maternal mental health. Women who reported experiencing physical violence from their partners were 1.75 times more likely to have major depression. This finding was consistence with other studies done across the world [15, 21, 24, 25]. There is serious consequences of physical violence on maternal well-being, emphasizing the need for comprehensive support and interventions for women experiencing domestic violence.

Furthermore, controlling behaviours from partners were strongly associated with major depression, with aPR of 3.60. Controlling behaviours can undermine women’s autonomy and sense of self-worth, contributing to increased stress and depression during pregnancy.

Conversely, the study identified a protective factor against major depression: having a wanted pregnancy. Women who reported their pregnancies as wanted experienced a 46% reduction in the likelihood of major depression. This was also reported in other studies done central Uganda, Ethiopia and China [12, 21, 26]. This finding emphasizes the importance of reproductive autonomy and emotional preparedness in mitigating the risk of maternal depression during pregnancy.

### Strengths and limitations of the study

Its cross-sectional design, which precludes establishing causality between the identified factors and MD. Longitudinal studies would provide deeper insights into the temporal relationships and potential pathways linking these factors to maternal mental health outcomes. Additionally, the study’s reliance on self-report measures for sensitive topics such as IPV and mental health may introduce reporting bias, affecting the accuracy of the findings.

## Conclusions

Major Depression affects nearly one-third of pregnant women in Northern Uganda. Major Depression is more prevalent among women with co-wives, in arranged marriages, with unplanned pregnancies, whose partners had low income, who experienced physical violence or controlling behaviours from a partner. These findings highlight the urgent need for targeted interventions, including prevention, screening, and treatment services for Major Depression within Antenatal Care clinics. Implementing such measures is crucial to improving maternal, foetal, and neonatal health outcomes in the region.

## Electronic supplementary material

Below is the link to the electronic supplementary material.

Supplementary Material 1

## Data Availability

All relevant data are within the manuscript and its supporting information files. Questionnaire has been attached as supplementary file Data are available upon reasonable request from the first author.

## Abbreviations

ANC: Ante-natal care
ECSACOG: East central and southern african college of obstetrics and gynaecology
GBV: Gender based violence
GUREC: Gulu research ethical committee
GRRH: Gulu regional referral hospital
MD: Major depression
PNC: Post-natal care
PR: Prevalence ratio
SSA: Sub-saharan Africa
SD: Standard deviation
WHO: World health organisation
