# Supplementary material for: Major depression among pregnant women attending a tertiary teaching hospital in Northern Uganda assessed using DSM-V criteria
Source: BMC Pregnancy Childbirth. 2025 Apr 26;25:504. doi: 10.1186/s12884-025-07618-9 (PMC12032744; doi:10.1186/s12884-025-07618-9)
Supplement: Supplementary file 1 — Supplementary Material 1 [file 12884_2025_7618_MOESM1_ESM.pdf]

Version 3.0

Questionnaire

Serial Number:

Date :

Section A: Socio-demographic characteristic of the participants

1. Age (in completed years)

   ----

2. What is your highest level of education?
   a) None
   b) Lower Primary
   c) Upper Primary
   d) O-level
   e) A-level
   f) Tertiary
3. Where do you stay?

Village………………………………

Parish……………………………….

Sub-county…………………………

County…………………………….

District…………………………….

4. What is your marital status?
   a) Never married.
   b) In union (married or cohabiting)
   c) Divorced
   d) Windowed
5. At what age did you get married?

   ……………………………………………………….

6. At what age did you first have sex?

……………………………………………………….

7. What type of marriage?
    a) Love marriage
    b) Arranged married.
8. What is your employment status?
    a) Employed
    b) Unemployed
9. If employed, what is your occupation?
   …………………………………………………………………………
10. What is your estimated monthly income?
   ………………………………………………………………………….. Ugandan shillings
11. How many weeks is your pregnancy?
   ………………………………………………………………………… weeks
12. Was the pregnancy intended?
    a) Yes
    b) No
13. Do both of you (you and your husband) wanted the pregnancy?
    a) Yes
    b) Only me
    c) Only the man
14. What is your partner's level of education?
    a) None
    b) Lower primary
    c) upper primary
    d) O level
    e) A level
    f) Tertiary
15. What is your husband employment status?
    a) Employed
    b) Unemployed
16. What is your husband estimated monthly income?
   ………………………………………………………………………… Ugandan shillings
17. Does your husband have other wife or wives?

a) Yes

b) No

a) Sister/brother

18. How is the relationship between you and your husband`s family?

a) Very good

b) Good

c) Fair

d) Poor

e) Very poor

19. How many children do you have? ……………………………………………………..

20. Have you ever used contraception?

a) Yes

b) No

21. Are you currently using contraceptive?

a) Yes

b) No

22. What is your HIV status?

a) Negative

b) Positive

23. Does your husband take alcohol?

a) Yes

b) No

Section B: Gender-based violence screening tool
(*Use a No/Yes response to answer the following questions*)

No	Question	Current pregnancy	Past pregnancy	When not pregnant
**Physical violence by an intimate partner**				
1	Was slapped or had something thrown at her that could hurt her.			
2	Was pushed or shoved.			
3	Was hit with a fist or something else could hurt.			
4	Was choked or burnt on purpose			

5	The perpetrator threatened to use or used a gun, knife, or other weapons against her			
6	Was punched or kicked in the abdomen while pregnant			
7	If yes to any of the above, was violence less, the same, or better than before the current pregnancy?			
**Sexual violence by an intimate partner**				
8	Was physically forced to have sexual intercourse when she did not want to			
9	Had sexual intercourse when she did not want to because she was afraid of what her partner might do			
10	Was forced to do something sexual that she found degrading or humiliating			
**Emotional violence by an intimate partner**				
11	Was insulted or made to feel bad about herself			
12	Was belittled or humiliated in front of other people			
13	The perpetrator had done things to scare or intimidate her on purpose by the way he looked at her by yelling or smashing things			
14	The perpetrator had threatened to hurt someone she cared about			
**Economic violence**				
15	Destroyed my property intentionally			
16	Restricted me from accessing financial resources			
17	Restricted me from working			
18	Restricted me from studying			
19	Did not provide for me or pregnancy need			
**Controlling behaviours by an intimate partner**				
20	He tried to keep her from seeing friends			
21	He tried to restrict contact with her family of birth			
22	He insisted on knowing where she was always			
23	He ignored her and treated her indifferently			
24	He got angry if she spoke with another man			
25	He was often suspicious that she was unfaithful			
26	He expected her to ask permission before seeking healthcare			

Section C: Maternal major depression assessment

**No**	**Question**	No	Yes
1	For the past weeks, were you depressed or down, or felt sad, empty, or hopeless most of the day, nearly every day?		
2	In the past two weeks, were you much less interested in most things or much less able to enjoy the things you used to enjoy, most of the time?		
	**Over the two weeks, when you felt depressed or uninterested:**		
3.	Was your appetite decreased or increased nearly every day? Did your weight decrease or increase without trying intentionally?		

4.	Did you have trouble sleeping nearly every night (difficulty falling asleep, waking up in the middle of the night, early morning wakening or sleeping excessively)?		
5.	Did you talk or move more slowly than normal or were you fidgety, restless or having trouble sitting still almost every day? Did anyone notice this?		
6.	Did you feel tired or without energy almost every day?		
7.	Did you feel worthless or guilty almost every day?		
8.	Did you have difficulty concentrating or making decisions almost every day?		
9.	Did you repeatedly think about death, or have any thoughts of killing yourself, or have any thoughts of killing yourself, or have any intent or plan to kill yourself? Did you attempt suicide?		
10.	Do these symptoms cause significant distress or problems at home, at work, socially, in your relationships, or in some other way, and are they a change from previous functioning?		
